# The Physiological Limits of Motor Speed

**Published:** 1902-09-27

**Authors:** 


### The Hospital

A Journal of

The Medical Sciences and Hospital Administration.

Vol. XXXII.—No. 835. Edited by Sir Henry Burdett, K.C.B., and Solomon C. Smith, M.D., M.R.C.P. [September 27, 1902.

### The Physiological Limits of Motor Speed.

We are by no means desirous of entering into the legal questions involved in what is now spoken of as the motor problem, nor do we feel competent, in view of the different opinions which find expression even among motorists themselves, to say what is a safe or what is an unsafe speed, at any rate so far as concerns the motorist and his companions; but there are certain considerations, mainly physiological, which make us very certain that whatever may happen to the statutes which at present aim at restraining the rate at which motors are driven, the speed therein sanctioned is well up to, if not beyond, that which will in practice be allowed by the magistrates on a large number of English roads—that is, if the safety of the ordinary users of the roads is to be considered. It may well be admitted that with fairly straight and wide roads, free from crossings, and with borders unbroken by openings into fields or farms or houses, especially if provided with footpaths, a high speed may with safety be indulged in when there are no other travellers in sight. But these are conditions which in English roads are very rarely met with. Everyone who has had much experience in driving will know the frequency with which the unexpected happens—the pig that suddenly turns round, the child that runs out of an entry, the girl apparently quite safely engaged playing battledore, who, with eyes fixed upon her shuttlecock, runs straight under the horse's feet, the deaf old woman or the unobservant dreamer who steps off the causeway just in front of the carriage. These are but examples, and it is to be noted that all these good people, even the pig, are well within their rights in their use of the road. From the motorist's point of view, when his horn is sounded they ought no doubt to clear out of the way. But that is not the law, for they have as much right in the road as he has. Thus it comes about that the speed which may safely be indulged in does not depend, as he would have us believe, upon the wonderful power of pulling up which he possesses, nor upon his marvellous skill in steering, but must always be regulated to a large extent by the power of the other people to get out of the way, an affair of the physiological activity of their nervous systems. The skill of the driver is an important factor, of course, and if all people acted exactly alike under similar circumstances would doubtless be the main factor in the case; indeed, if one could feel assured that on the sound of the horn every living thing would stand rigid until the machine had gone by, much might be trusted to the skill of the motorists. But in view of the extreme variability in alertness of mind and of body which is to be found among the other users of the road, and of the still greater uncertainty as to the direction in which such alertness as they may possess will show itself, we come to this, that the speed which may safely be allowed upon the public roads has but little relation to the capacity of the machines employed, and must be decided rather by the ordinary average rapidity with which the ordinary average users of the road can, as the physiologists describe it, "solve a dilemma." Naturally this "reaction time," as it is called, is ruled partly by the activity of a man's mental or nervous processes, and partly by the complexity of the dilemma—a complexity, be it stated, which is greatly added to by the evil habit, so much practised by cyclists and motorists, of passing on the wrong side of vehicles which they overtake. In practice the "reaction time" is also much influenced by other factors—the deficient muscular powers of elderly folk, the loads which people may be carrying or wheeling, and the partial inhibition of neuromuscular activity which falls upon some people when suddenly exposed to peril, or what they take to be peril. In considering, then, what may be regarded as a safe and permissible speed for travellers upon public roads, we have not to consider chiefly, or even very largely, the powers of the machine or the skill of the driver, but rather the average "reaction time" of the ordinary users of the road. Thus we cannot entirely agree with those who consider that with improved machines and more skilful driving a large increase of speed will become permissible. So far as the machines increase their power of "pulling up" they will become safer, while with improvement in tires, steering at high speed may become less perilous, and so, again, safety may be increased; but, after all, the limit of speed must to a very large extent depend upon the "reaction time" or alertness of the average users of the road, among whom we must always count upon the presence of a fair proportion of old people, children, and fools. Thoroughly as we admit the possibility of a good motor running safely at a very high speed along a clear course, we greatly doubt whether the motorists will gain much by the abolition of the 12 miles an hour rule—which, indeed, as it stands, is too often regarded as a licence for overriding the common law in regard to driving to the common danger.

